# Wearable Ultrasound Devices for Therapeutic Applications

**DOI:** 10.1007/s40820-025-01890-2

**Published:** 2025-08-26

**Authors:** Sicheng Chen, Qunle Ouyang, Xuanbo Miao, Feng Zhang, Zehua Chen, Xiaoyan Qian, Jinwei Xie, Zheng Yan

**Affiliations:** 1 Department of Chemical and Biomedical Engineering, University of Missouri, Columbia, MO USA; 2 Department of Mechanical and Aerospace Engineering, University of Missouri, Columbia, MO USA; 3 Department of Surgery-Transplant and Mary and Dick Holland Regenerative Medicine Program, University of Nebraska Medical Center, Omaha, NE USA; 4 NextGen Precision Health, University of Missouri, Columbia, MO USA

**Keywords:** Wearable ultrasound devices, Drug delivery, Tissue regeneration, Closed-loop therapy, Neurorehabilitation

## Abstract

Wearable ultrasound devices represent a transformative advancement in therapeutic applications, offering noninvasive, continuous, and targeted treatment for deep tissues. These systems leverage flexible materials (e.g., piezoelectric composites, biodegradable polymers) and conformable designs to enable stable integration with dynamic anatomical surfaces. Key innovations include ultrasound-enhanced drug delivery through cavitation-mediated transdermal penetration, accelerated tissue regeneration via mechanical and electrical stimulation, and precise neuromodulation using focused acoustic waves. Recent developments demonstrate wireless operation, real-time monitoring, and closed-loop therapy, facilitated by energy-efficient transducers and AI-driven adaptive control. Despite progress, challenges persist in material durability, clinical validation, and scalable manufacturing. Future directions highlight the integration of nanomaterials, 3D-printed architectures, and multimodal sensing for personalized medicine. This technology holds significant potential to redefine chronic disease management, postoperative recovery, and neurorehabilitation, bridging the gap between clinical and home-based care.

## Highlights

- Flexible ultrasound devices enable deep-tissue therapy via conformable designs, overcoming limitations of rigid systems for continuous monitoring and treatment.
- Cavitation-enhanced drug delivery and neuromodulation demonstrate noninvasive, targeted interventions for chronic diseases and neural disorders.
- Wireless, AI-integrated platforms pave the way for personalized, adaptive therapeutics in home-based and clinical settings.

## Graphical abstract

**Figure:**
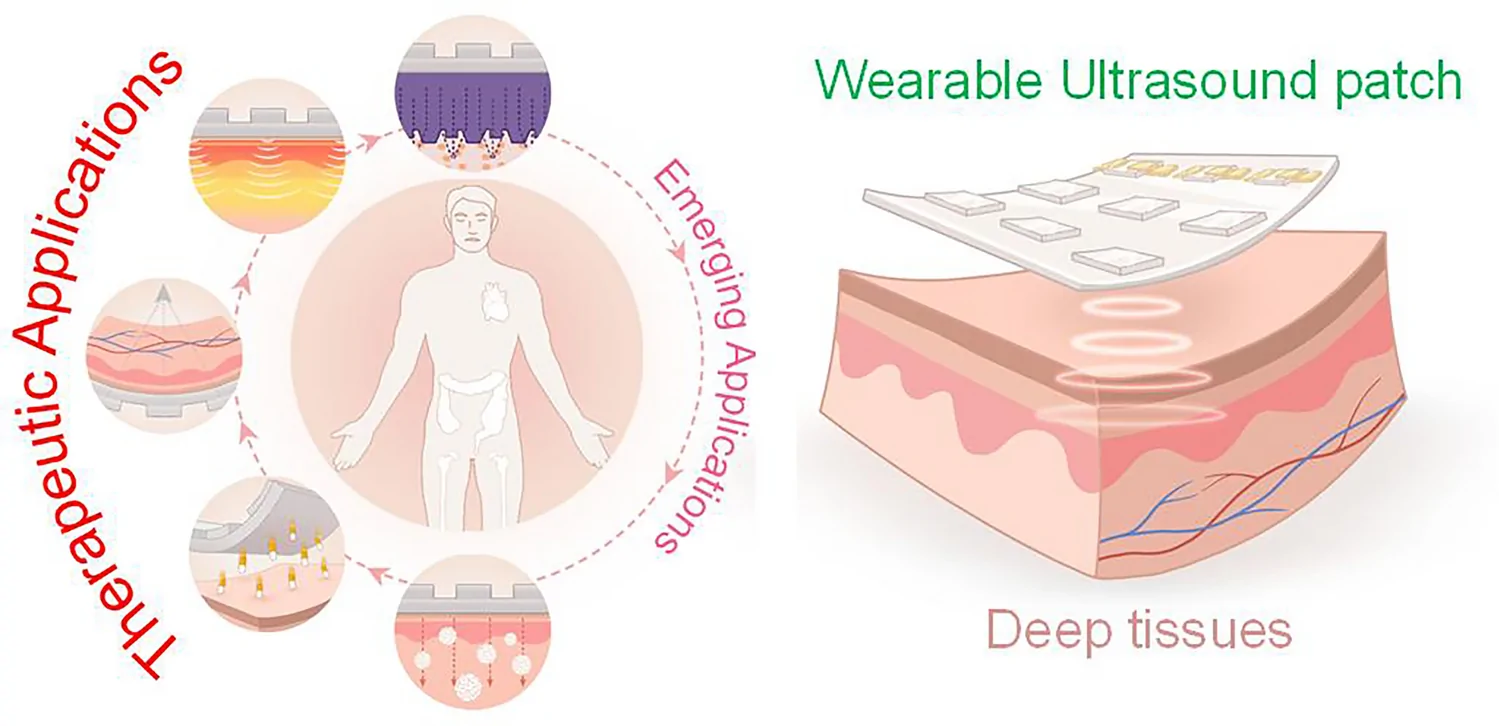

## Introduction

**Fig. 1:**
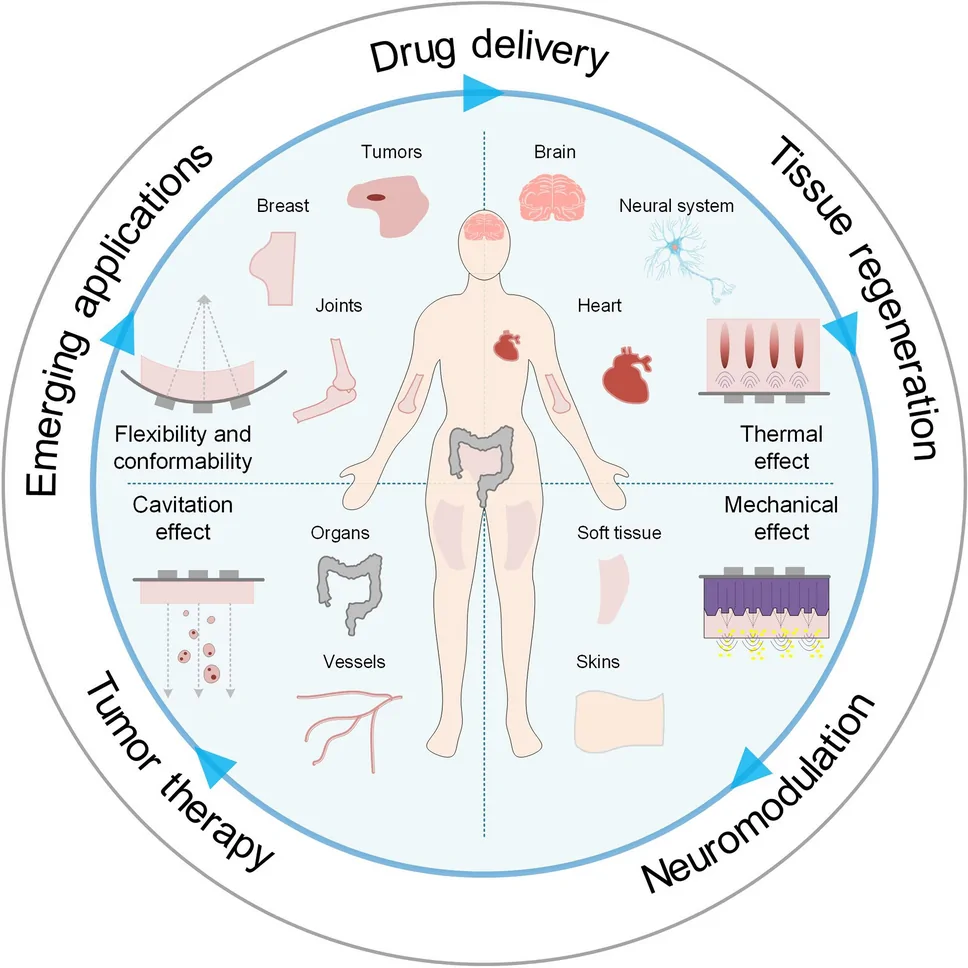
Summary of ultrasound technology applications across diverse therapeutic purposes, including drug delivery, tissue regeneration, neuromodulation, and tumor therapy. Wearable biodevices demonstrate emerging applications tailored to specific organs

With the rapidly increasing healthcare demands, aging populations, and the growing necessity for continuous diseases monitoring, global healthcare systems have faced unprecedented challenges due to a lack of efficient, accessible, reliable diagnostic and therapeutic solutions [1–3]. Increasing individuals have recognized that most of the diseases are from physical inactivity, inadequate nutrition, and unhealthy lifestyle. Therefore, accomplish the functions of continuous monitoring and real-time analysis of the vital signals which are critically important to prevent diseases and safeguarding the individual health. However, traditional healthcare solutions often require periodic hospital visits, reliance on bulky equipment, and substantial healthcare infrastructure, resulting in patient and potential delays in healthcare delivery. These drawbacks high light the urgent need for medical devices that empower individuals to conveniently manage their own health conditions. In response to this demand, wearable biodevices have emerged as promising alternatives to traditional devices, offering advantages such as portability, wearability, remote access, and timeliness [4, 5]. Providing effective approaches to regularly and continuously monitor a wide range of physiological signals (e.g., blood pressure [6], pulse [7], temperature [8]), thermal data (e.g., thermal con ductivity, temperature distribution [9]), mechanical cues (e.g., strain [10], pressure [11, 12]), electrophysiological (e.g., electrocardiogram (ECG) [13, 14], electroencephalo gram (EEG) [15], electromyography (EMG) [16], electrooculogram (EOG) [17]), and biochemical signals (biomarkers in body fluids [18–21]). Despite their promise, current wearable technologies rely primarily on optical, electrical, or chemicals which limit their penetration depth and capacity for drug delivery [22–24]. These constraints leave deeper tissue such as muscle layers or visceral organs largely unmonitored, a critical shortcoming for early-stage disease. Ultrasound technology uniquely complements wearable platforms due to its intrinsic ability to penetrate deeply into biological tissues while providing real-time imaging and feedback without ionizing radiation. Ultrasound employs mechanical waves at frequencies typically ranging from 20 kHz to 50 MHz, making it an ideal modality for deep-tissue diagnostics and therapeutic applications [25–28]. Custom ultrasound device designs typically involve bulky, rigid, and operator-dependent systems that cannot comfortably interface with the human body or adequately adapt to anatomical irregularities, which are unsuitable for prolonged use or continuous monitoring, severely limiting their practical applications in personalized healthcare. Recently developed flexible and wearable ultrasound systems have overcome these constraints by adaptively conforming to anatomical surfaces, enabling stable, high-resolution imaging, accurate long-term physiological monitoring, treatment, and comfortable, user-friendly integration with patients [2, 29–31]. These wearable ultrasound devices offer significant improvements in patient compliance and comfort, reduce reliance on highly skilled operators, and pave the way for personalized healthcare solutions that support real-time clinical decision-making and patient self-management. In this review, we focus on the therapeutic applications of wearable ultrasound. Recent advancements in wearable ultrasound therapeutics have demonstrated broad applicability across multiple medical domains (Fig. 1). Specifically, flexible ultrasound devices have been successfully utilized in precise drug delivery, enabling controlled, targeted, and transdermal pharmaceutical applications to challenging anatomical sites like breast tissue [32, 33], joints [34, 35], and internal organs [36, 37] through ultrasound-induced cavitation effects. These devices demonstrate significant potential in promoting tissue regeneration, exhibiting therapeutic effects comparable to those achieved by conventional regenerative medicine approaches [38–41]. Furthermore, wearable ultrasound has shown exceptional promise in the realm of neuromodulation, providing non-invasive neural stimulation techniques capable of precisely modulating nerve functions in deep tissues [42–45]. Finally, wearable ultrasound devices continue to evolve into emerging therapeutic applications, driven by innovative materials, enhanced device flexibility, and integration with multimodal sensing and stimulation platforms. By synthesizing recent breakthroughs in materials science, mechanical flexibility, and precise acoustic control, conformable ultrasound electronics represent a transformative direction in wearable therapeutics.

## Fundamentals of Ultrasound Therapy

**Fig. 2:**
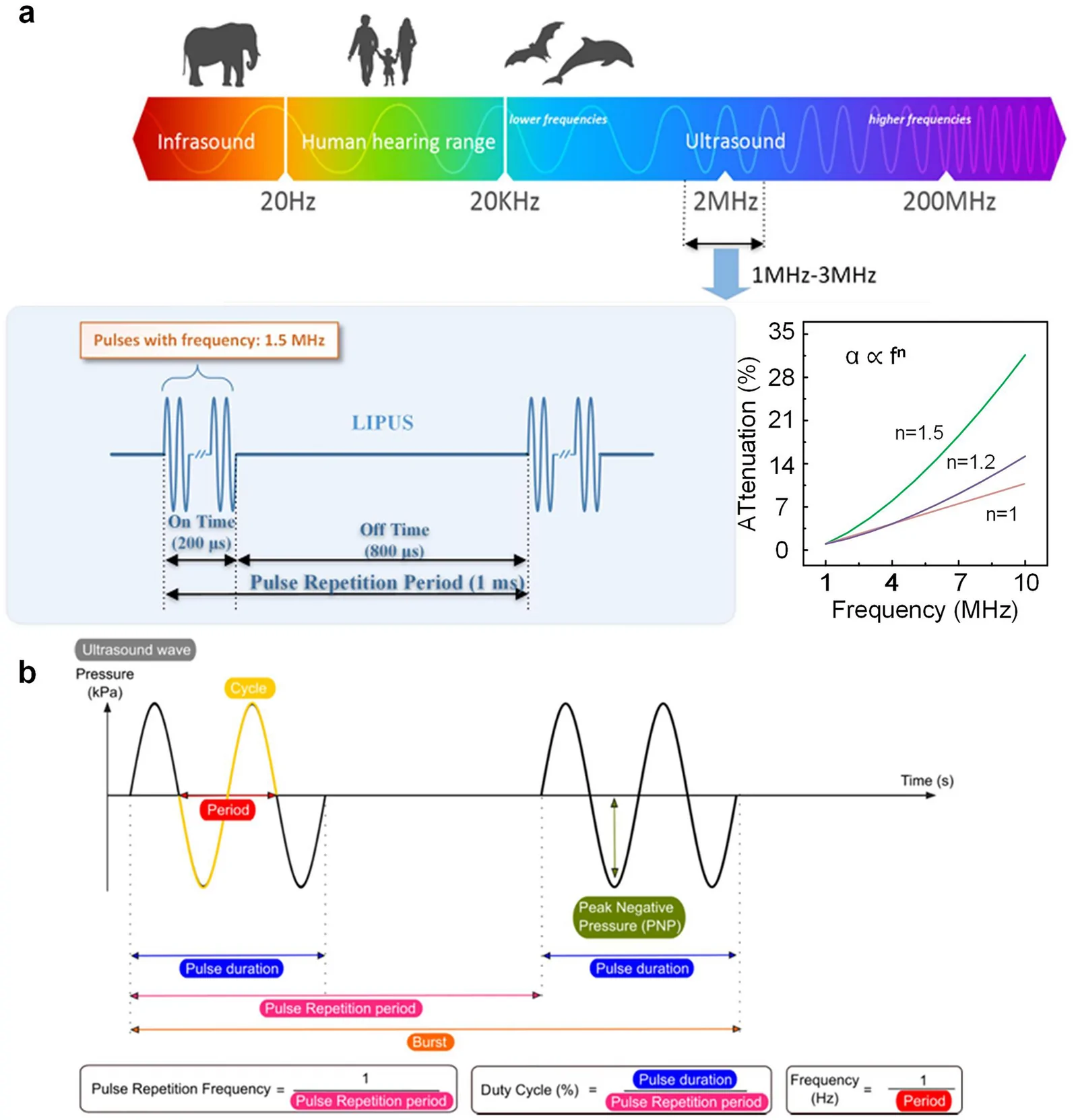
**a** The range of ultrasonic wave frequencies and the relationship between attenuation coefficient and frequency [54]. Copyright 2018, IEEE. **b** Overview of relevant ultrasound wave characteristics. The ultrasound wave characteristics strongly define microbubble cavitation behavior and biological outcome and are therefore important to report. Reproduced with permission [56]. Copyright 2021, Elsevier

Ultrasound is a high-frequency sound wave that propagates through a medium in the form of mechanical vibrations [46, 47]. It is typically generated and detected using piezoelectric crystals, which convert electrical energy into mechanical waves through the inverse piezoelectric effect [48–51]. Since ultrasound operates at frequencies beyond the human audible range (typically hundreds of KHz to several MHz), it offers significant advantages in medical imaging and therapeutic applications due to its ability to penetrate tissues while maintaining high spatial resolution. The way ultrasound is transmitted plays a fundamental role in its applications. Continuous wave ultrasound emits sound waves uninterruptedly, making it ideal for Doppler-based blood flow measurements that require continuous frequency shift detection [52, 53]. However, because the waves never pause, imaging depth cannot be determined, limiting its usefulness in imaging. In contrast, pulsed wave ultrasound, widely used in both imaging and therapy, alternates between short bursts of energy and echo reception, allowing depth calculations based on time delays and enabling high-resolution imaging and precise targeting (Fig. 2a) [52, 54, 55]. The relationship between attenuation and frequency is a critical aspect of ultrasonic wave propagation, where the attenuation coefficient (*α*) serves as a function of frequency (*f*) for the relevant medium. The inserted plot confirms the expected trend of increasing attenuation with higher frequencies, following a power-law dependence (*α* is proportional to *fⁿ*, where *n* ranges from 1 to 2 for typical biological tissues). Each pulse consists of multiple cycles, which define pulse duration, whereas the interval between pulses-called the pulse repetition period-determines how often pulses are emitted (Fig. 2b) [56]. These acoustic parameters ultimately govern the biological effects of the propagating ultrasound waves. Thus, a thorough understanding of ultrasound fundamentals-including wave generation, propagation properties, and critical operational parameters is essential to optimize its therapeutic applications [28]. Furthermore, wearable ultrasound devices have revolutionized clinical practice by leveraging three well-characterized biophysical mechanisms—precisely controlled thermal elevation, optimized cavitation dynamics, and tailored mechanical stresses. These portable systems enable unprecedented therapeutic capabilities, including continuous blood–brain barrier opening for neurodegenerative disease treatment, real-time monitoring and adjustment of thermal doses in tumor therapy, and personalized rehabilitation protocols for musculoskeletal disorders. Their unique combination of sustained operation, patient-specific adaptability, and closed-loop feedback has addressed critical limitations of conventional ultrasound systems, particularly in chronic conditions requiring long-term intervention, chronic wounds, and metastatic cancers [57–61].

### Principle of Ultrasound

Ultrasound therapy relies on the controlled generation and transmission of mechanical waves through biological tissues. These waves are typically produced by piezo electric transducers, which convert alternating electrical signals into mechanical vibrations via materials, such as lead zirconate titanate or advanced relaxor ferroelectrics [62–65]. When stimulated, these materials oscillate at high frequencies, creating acoustic pressure waves that travel through soft tissues at tissues-specific sound speeds-approximately 1540 m s^−1^ in many soft tissues [66]. Mathematically, acoustic wave propagation is often described by the acoustic wave equation [67]:

1 $$\nabla^{2} p - \frac{1}{{c^{2} }}\frac{{\partial^{2} p}}{{\partial t^{2} }} = 0$$

where *p* is the acoustic pressure, *c* is the tissue-specific sound speed, and α is the frequency-dependent attenuation coefficient of the tissue. As ultrasound waves traverse biological tissues, several interrelated parameters collectively govern the therapeutic outcomes they can achieve. Frequency is pivotal, as it dictates both penetration depth and spatial resolution-lower frequencies can reach deeper tissues but provide coarser resolution, whereas higher frequencies offer sharper resolution at shallower depths [48, 68]. Equally important is intensity, which describes the amount of acoustic energy delivered per unit area; maintaining this within safe ranges ensures effective stimulation or ablation without injuring surrounding tissues [69–71]. Pulse parameters including repetition frequency and duty cycle-further modulate the total energy dose, influencing the balance between thermal and non-thermal effects as well as overall safety [72]. Finally, focus control enables precise localization of acoustic energy in the targeted region [73–75]. By carefully tuning frequency, intensity, pulse parameters and focus, clinicians and researchers can optimize ultrasound therapy for a wide array of clinical applications, balancing safety with efficacy in both diagnostic and therapeutic.

### Mechanisms of Therapeutic Ultrasound

Therapeutic ultrasound exerts its efficacy predominantly via thermal effects, cavitation, and mechanical stresses within biological tissues. When acoustic waves propagate through tissue, a portion of their energy is absorbed and converted to heat, raising local temperatures in a manner that varies with tissue composition [76, 77]. High-intensity focused ultrasound (HIFU) leverages this thermal buildup to ablate target tissues (e.g., tumors). In the focal area, tissue temperature increases rapidly, within seconds, up to 60 °C or higher, while lower yet still elevated temperatures can facilitate enhanced drug delivery by destabilizing thermosensitive carriers or altering tumor microenvironments [78–80]. In tandem, cavitation occurs when ultrasonic energy induces the formation, oscillation, and collapse of microbubbles in a liquid medium. Stable cavitation can generate fluid microstreaming that distorts cell membranes, whereas inertial cavitation produces violent bubble collapses, generating shock waves, microjets, or even transient free radicals that disrupt cells [81]. This phenomenon underpins various applications, from transdermal drug permeation and tumor ablation to localized blood–brain barrier opening [82–86]. As a mechanical wave, ultrasound can also deliver mechanical stresses through radiation force or bubble oscillations, thereby stimulating tissue regeneration, promoting angiogenesis, or modulating neuronal activity [87].

**Fig. 3:**
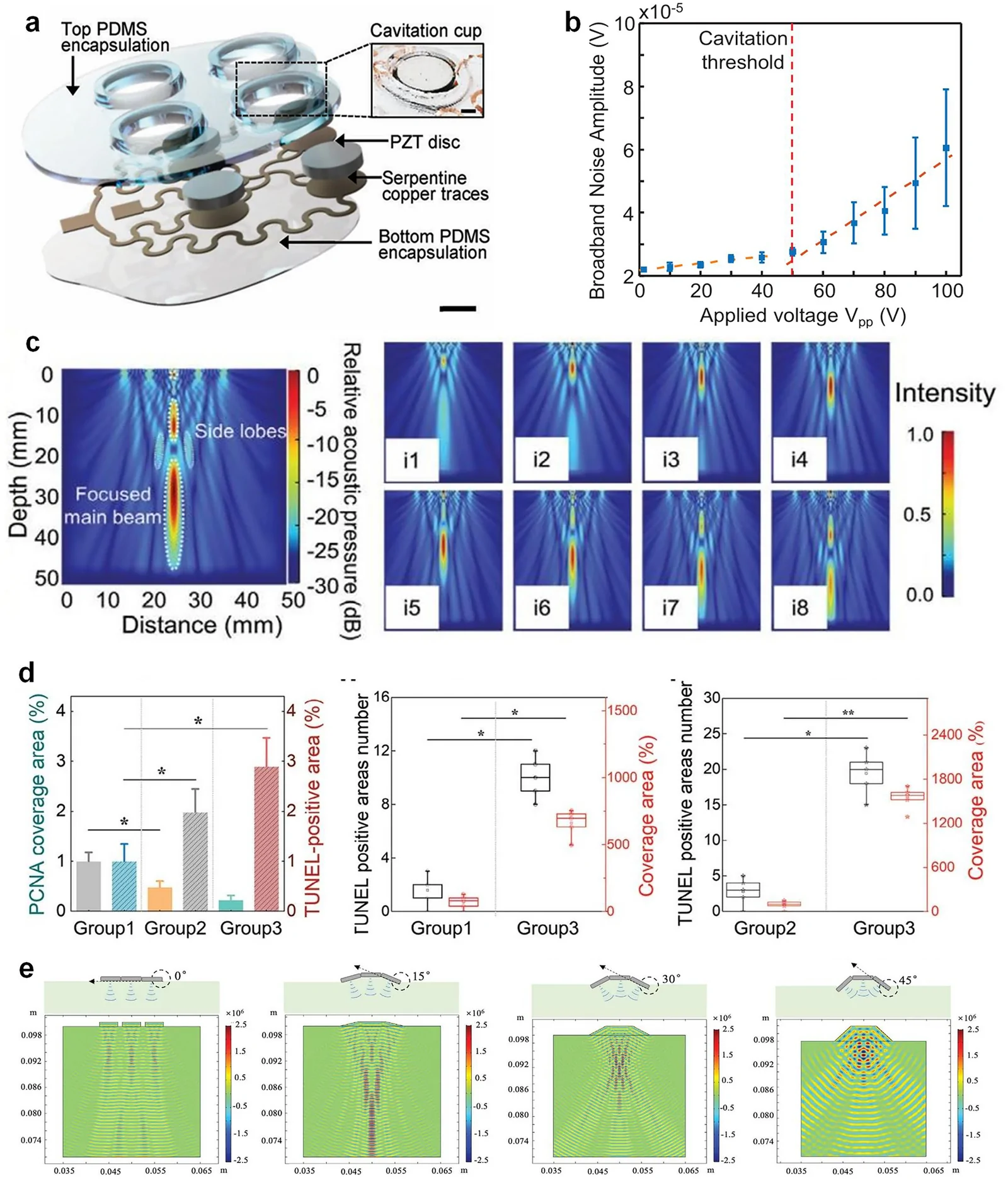
**a** Exploded view of the 2D-array of cUSP showing each constituent layer; scale bar: 5 mm (the inset shows the fluid cavity formed between the PZT-D element and skin; scale bar: 2 m. Reproduced with permission [88]. Copyright 2023, Wiley–VCH GmbH. **b** Broadband noise amplitude as a function of applied voltage to the PZT-D. Reproduced with permission [88]. Copyright 2023, Wiley–VCH GmbH. **c** Simulated acoustic emission profile of a piezoelectric material with a size of 0.2 × 0.2 mm^2^ (inset) with excellent beam directivity, focus, and penetration depth (> 40 m. Reproduced with permission [89]. Copyright 2024, Wiley–VCH GmbH. **d** Relative coverage percentages of PCNA- and TUNEL- positive areas. Data are expressed as mean ± SD (*n* = 5); The number of TUNEL- positive areas between group 1 and group 3; The number of TUNEL- positive areas between group 2 and group 3; The differences among groups were calculated using one- way ANOVA. *p < 0.05, **p < 0.01. Reproduced with permission [89]. Copyright 2024, Wiley–VCH GmbH. **e** Sound waves are distributed vertically along the direction of the array when the bending degree ranges from 0 to 45 degree. Reproduced with permission [90]. Copyright 2021, Wiley–VCH GmbH

Recent advances in wearable ultrasound technology have successfully translated these fundamental mechanisms—including thermal effects, cavitation, and mechanical stresses—into clinically effective therapeutic applications. For instance, Fig. 3a depicts a thin polymer substrate integrated with piezoelectric transducers (PZT), separated by a 1-mm annular gap to permit undamped radial oscillation [88]. This design enhances cavitation around the patch surface, enabling high-efficiency energy transfer for tasks like drug penetration and wound therapy. Exceeding a certain applied voltage (50 V) triggers a sharp rise in broadband noise, indicating the onset of robust inertial cavitation (Fig. 3b). Controlling ultrasound depth and focusing is equally critical: demonstrate a conformable wearable ultrasound (CWUS) patch whose phased elements produce a main beam and side lobes for spatially localized energy, potentially damaging tumor cells or improving tissue repair at depth (Fig. 3c, d) [89]. Flexibility is further highlighted in Fig. 3e, where the ultrasonic patch can bend to a radius under 5 mm, maintaining skin conformity [90]. In a flat state, the beam remains broadly vertical; when bent, the focal region shifts closer to the patch, concentrating energy at a targeted point in the underlying tissue. Such adaptability not only improves coupling and patient comfort compared to rigid probes but also enables adjustable treatment depths for diverse clinical needs-ranging from accelerating wound healing to temporarily disrupting barriers (e.g., skin or vasculature) for controlled substance delivery. Used 2 MHz low-frequency ultrasound in a flexible patch design can achieve transcranial penetration depths of 60–80 mm (comparable to clinical TCD devices) while maintaining a signal-to-noise ratio improvement of 40% over rigid probes due to better skull coupling [80]. As wearable ultrasound devices continue to evolve, the interplay of thermal, cavitation, and mechanical effects stands at the core of their therapeutic promise, enabling increasingly personalized, minimally invasive interventions in oncology, regenerative medicine, neuromodulation, and beyond.

## Evolution of Wearable Ultrasound Devices

For decades, ultrasound has been a cornerstone of clinical procedures due to its cost-effectiveness, noninvasiveness, safety, versatility, and convenience. The piezoelectric effect, involving the conversion of electrical signals into mechanical vibrations, is the predominant operating principle for actuating and sensing ultrasonic signals [91]. These conventional probes, composed of rigid piezoelectric materials such as lead zirconate titanate, encounter significant limitations, particularly in imaging curved anatomical regions such as elbows, knees, and the skull, as well as large areas such as the breast or abdomen. Additionally, conventional ultrasound devices have traditionally been confined to clinical settings, hindering long-term personalized monitoring and dynamic physiological assessments. In response to these limitations, the concept of wearable ultrasound-encompassing wearable, flexible, and soft ultrasound sensors, transducers, and patches has emerged and evolved rapidly since its inception prior to 2010 [92]. Next, we will describe the development of wearable ultrasound devices relies on several crucial innovations and components, including: (i) flexible technology that determine the overall mechanical properties of the device, which including the stretchable materials and stretchable electrical interconnects [93–96]. (ii) efficient energy management solutions ensuring device autonomy and portability [97–99], and (iii) advanced communication and control technologies facilitating real-time monitoring and adaptive therapeutic interventions [100–102].

### Flexible Technology

Wearable ultrasound devices demand high mechanical compliance to conform reliably to curved or irregular body surfaces. Achieving such adaptability requires not only the integration of soft and deformable substrates but also the use of structurally engineered conductors that remain robust under frequent bending and stretching. Recent advances have therefore focused on two key strategies: the development of flexible substrates with serpentine interconnects for stable electrical performance and the exploration of conductive polymers that offer intrinsic pliability [34, 103–106].

**Fig. 4:**
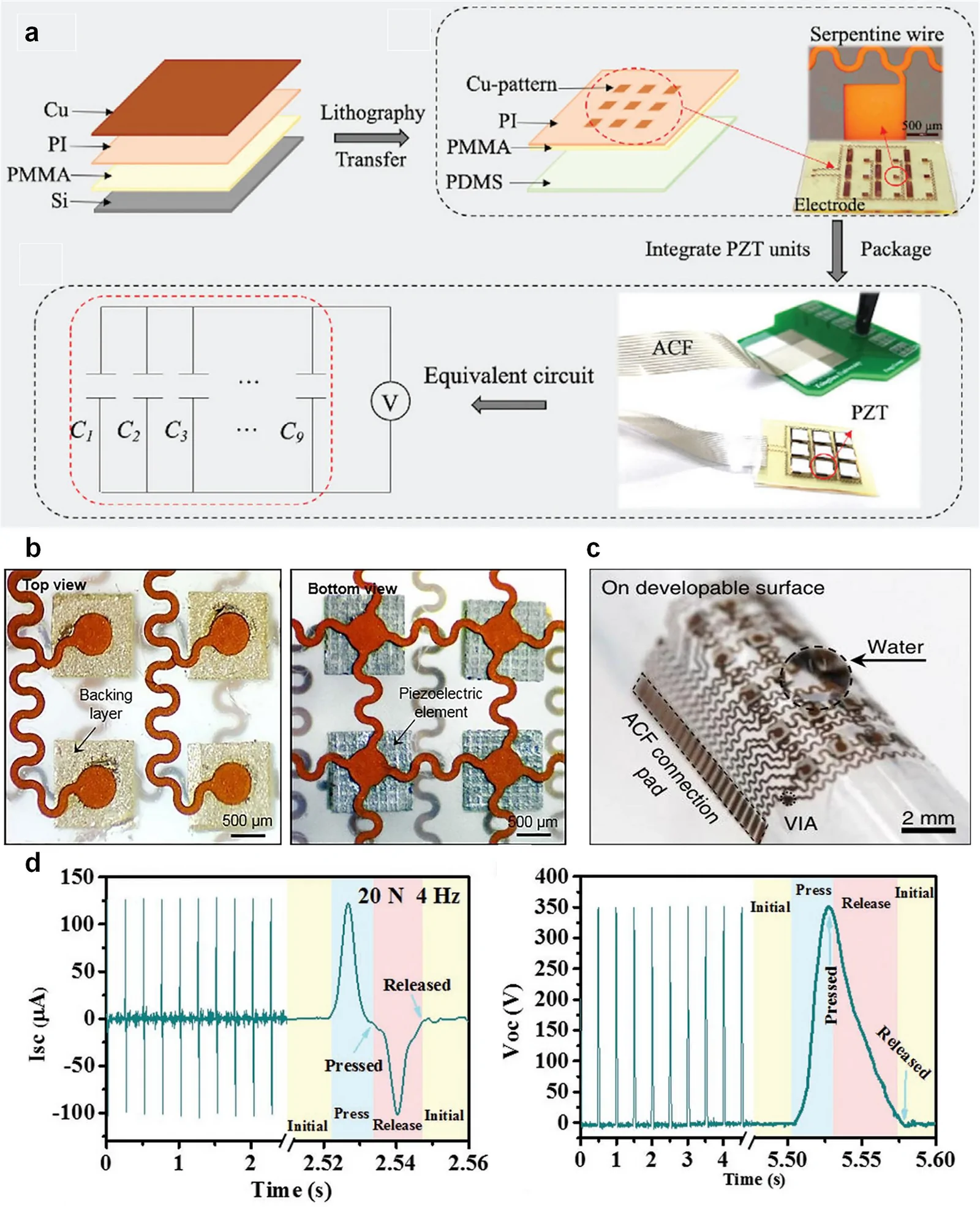
**a** PMMA, PI and Cu metal layers are spin-coated on the Si substrate. Lithography, etching and transfer printing are used to fabricate the flexible circuit. Piezoelectric ultrasonic units (PZT—4) are integrated into the flexible circuit, which is then packaged with a thin (≈200 μ hydrogel patch. Reproduced with permission [90]. Copyright 2021, Wiley–VCH GmbH. **b** Optical image (top view) of four elements, showing the morphology of the backing layers and top electrodes, and the optical image (bottom view) of four elements, showing the morphology of the piezoelectric material and bottom electrodes. Reproduced with permission [34]. Copyright 2018, AAAS. **c** Bent around a developable surface. Reproduced with permission [31]. Copyright 2018, Springer Nature. **d** Compression test of the hybrid NG: short circuit current and open circuit voltage. Reproduced with permission [118]. Copyright 2020, WILEY–VCH Verlag GmbH & Co. KGaA

Flexible substrates are fundamental in the fabrication of wearable ultrasound transducers, as they dictate the overall mechanical properties, conformability, and durability of the device. Various materials have been explored, including elastomers, soft polymer films, and ultrathin metallic foils. These materials are chosen for their intrinsic flexibility, elasticity, and ability to endure stretching and bending without failure. Among the most used elastomers, polydimethylsiloxane (PDMS) has gained significant attention due to its biocompatibility, high elasticity (capable of sustaining over 170% tensile strain), and excellent processability [106, 107]. PDMS functions both as a flexible substrate embedded with rigid piezoelectric elements and as an encapsulation layer for protection against environmental factors. Another widely used elastomer is Ecoflex, which possesses a lower Young’s modulus (~ 60 kPa) compared to PDMS (~ 1 MPa), making it suitable for applications requiring enhanced skin conformity and mechanical compliance [108]. Researchers have leveraged Ecoflex for fabricating fully flexible ultrasound transducers, embedding piezoelectric elements within its low-modulus matrix to achieve better adaptability to curved anatomical regions [109]. Additionally, polyurethane (PU) polymers are used for their excellent flexibility, resistance to fatigue, and superior acoustic damping properties. In some designs, PU rubber has been employed as an electrical insulator and mechanical protective layer for backing materials in ultrasound transducers [110, 111]. Other thermoplastic polymers, such as polyimide (PI) [112], polyethylene terephthalate (PET) [113, 114], and polyethylene naphthalate (PEN) [115], offer high mechanical robustness while maintaining transparency and flexibility. PI has demonstrated exceptional tensile strength, biocompatibility, and resilience to deformation, making it a viable option for fabricating micromachined ultrasound transducer (PMUT) arrays. For example, a flexible ultrasound patch is fabricated using a multi-layered structure with a soft PDMS substrate and an island-bridge electrode layout for enhanced flexibility (Fig. 4a) [90]. The process begins with a PMMA-coated silicon substrate, followed by a PI support layer and a patterned metallic electrode. Piezoelectric units are bonded onto rigid islands, interconnected by serpentine conductive traces that stretch and bend without breaking. Anisotropic conductive film (ACF) routes signals for uniform electrical excitation. The patch conforms to curved surfaces with a bending radius under 5 mm, ensuring effective acoustic coupling. Except for stretchable materials, the structure like the island-bridge design prevents strain accumulation on the rigid piezoelectric elements, the actual signal transmission and energy efficiency rely heavily on the integration of high-performance piezoelectric materials and robust interconnect structures. Serpentine-shaped conductive interconnects have emerged as a key innovation in flexible electronics, offering stretchability and mechanical resilience while maintaining stable electrical conductivity [116]. Traditional metal thin films or foils are brittle and prone to cracking under deformation, but by engineering their geometry into serpentine structures, they can undergo significant mechanical strain without failure. These serpentine interconnects act as electrical pathways, linking rigid transducer islands while allowing for extensive stretching and bending. Photolithography and laser cutting techniques are commonly employed to fabricate these serpentine-structured interconnects, ensuring precise patterning and mechanical compliance [103, 117].

**Fig. 5:**
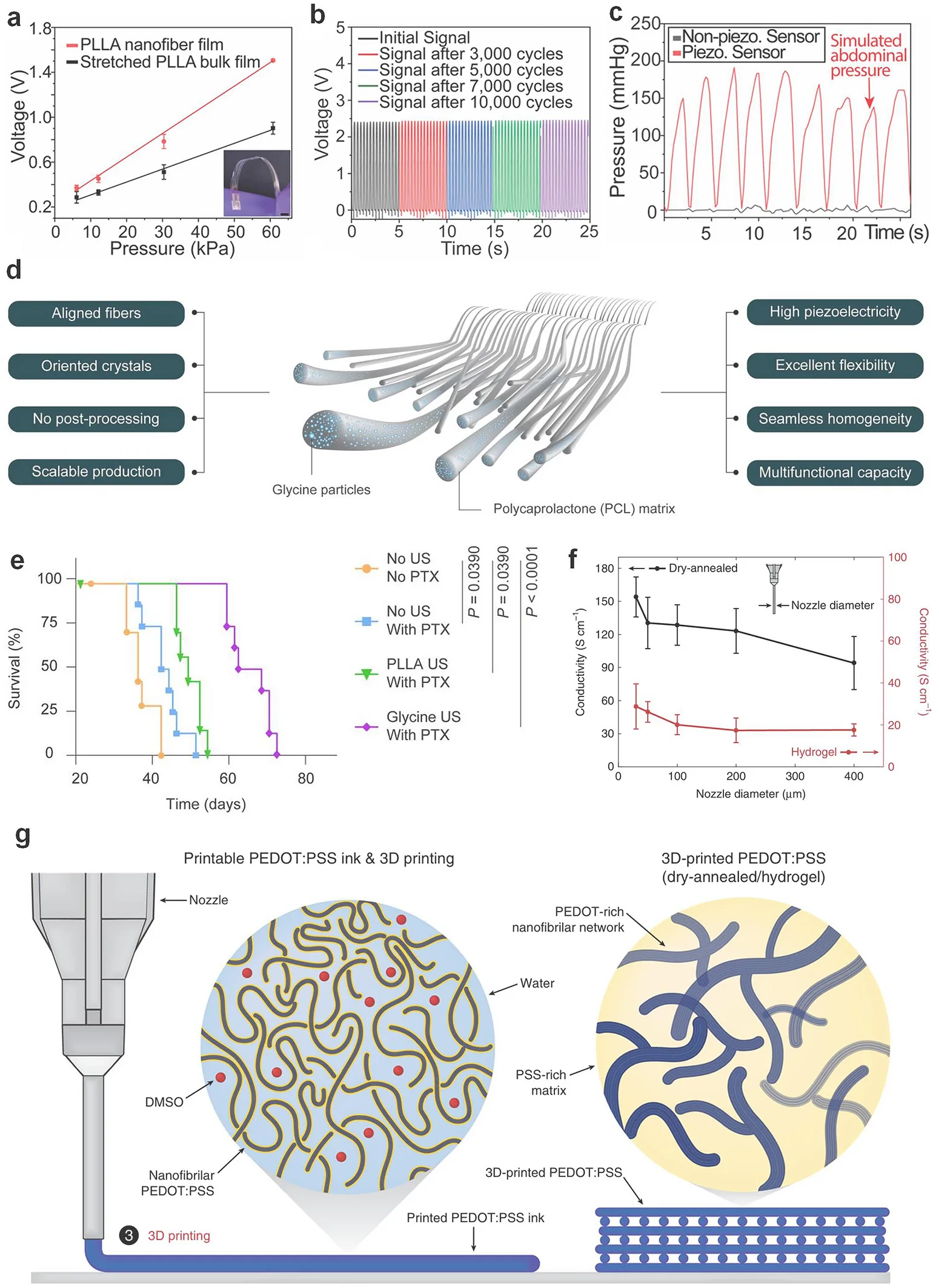
**a** Comparison of calibration curves for a biodegradable sensor using stretched, bulk piezo PLLA film (bl and a 4,000 rpm electrospun PLLA nanofiber film (red). Reproduced with permission [120]. Copyright 2019, PNAS. **b** Output from a charge amplifying circuit connected to a 4,000 rpm electrospun, biodegradable PLLA sensor that is subjected to 10,000 cycles of a 10-N force. Reproduced with permission [120]. Copyright 2019, PNAS. **c** Comparison of the simulated abdominal pressure signals, wirelessly recorded from an implanted biodegradable PLLA nanofiber sensor using a 300 rpm negative control (bland a 4,000 rpm film). Reproduced with permission [120]. Copyright 2019, PNAS. **d** Schematic illustration of glycine-PCL nanofibers. Reproduced with permission [121]. Copyright 2023, AAAS. **e** Kaplan–Meier survival of animals receiving different treatments (*n* = 8, log-rank test). Reproduced with permission [121]. Copyright 2023, AAAS. **f** Conductivity as a function of nozzle diameter for 3D-printed conducting polymers in dry and hydrogel states. Reproduced with permission [122]. Copyright 2020, Springer Nature. **g** 3D-printed conducting polymers can be converted into a pure PEDOT:PSS both in dry and hydrogel states by dry-annealing and subsequent swelling in wet environment, respectively. Reproduced with permission [122]. Copyright 2020, Springer Nature

Beyond flexible substrates, piezoelectric 1–3 composites have become the preferred choice for wearable ultrasound transducers owing to their enhanced electromechanical coupling properties compared to conventional isotropic PZT materials. In this structure, piezoelectric pillars embedded in an epoxy matrix suppress lateral vibrations while enhancing longitudinal wave propagation, maximizing energy conversion and ultrasound output. To improve acoustic matching and signal fidelity, a silver-epoxy backing layer dampens vibrations, broadens bandwidth, and ensures strong electrical connectivity. With an acoustic impedance (~ 20 MRayl) closely matching human tissue (~ 1.5 MRayl), no additional matching layer is needed (Fig. 4b) [34]. To ensure long-term stability and skin-conformal integration, the entire device is encapsulated within a thin silicone elastomer, as depicted in Fig. 4c [31]. This elastomeric encapsulation fulfills several critical functions. Mechanical protection is achieved through structural reinforcement, which prevents mechanical failure under repeated bending, stretching, and twisting. Acoustic performance optimization is enabled by the ultrathin (15 μm) elastomer layer, which maintains a balance between mechanical flexibility and efficient ultrasound transmission. Owing to its soft mechanical properties, the encapsulated ultrasound patch can conform to both developable (smooth) and non-developable (complex) anatomical surfaces. This conformability, combined with its durability under mechanical deformation, underscores the device’s robustness and suitability for continuous, skin-integrated operation in dynamic, real-world environments. Recent advancements in integrate conductive polymers, flexible and biodegradable piezoelectric materials have enabled the development of next-generation ultrasonic devices that are not only mechanically adaptable but also capable of controlled degradation within biological environments. One significant breakthrough in biodegradable piezoelectric materials has been the use of poly (L-lactic acid) (PLLA) for the fabrication of nanofibers that exhibit controlled and stable piezoelectric performance (Fig. 4d) [118, 119]. PLLA has been widely utilized in medical applications due to its biocompatibility and mechanical durability. The development of biodegradable PLLA-based nanofibers through electrospinning has demonstrated their potential for a variety of biomedical applications, including biodegradable pressure sensors for monitoring physiological pressures, ultrasonic transducers for blood–brain barrier opening to enhance non-invasive drug delivery, and implantable medical ultrasound devices that degrade naturally after their functional lifespan (Fig. 5a–c) [120]. Beyond PLLA, glycine-polycaprolactone (PCL) piezoelectric composites have emerged as another promising material for biodegradable ultrasound transducers (Fig. 5d) [121]. The ultrasonic device, comprising glycine-PCL nanofibers, molybdenum (Mo) electrodes, and polylactic acid (PLA) encapsulation layers, has demonstrated excellent performance in biomedical applications. The selection of Mo and PLA, both extensively used in FDA-approved biodegradable implants, ensures the safety and reliability of these transducers for in vivo applications. Unlike conventional ultrasound transducers, which require surgical extraction, glycine-PCL-based devices degrade naturally over time, thereby eliminating the need for secondary procedures. To evaluate the efficacy of glycine-PCL nanofibers in ultrasound generation, their performance has been compared with other biodegradable piezoelectric materials, including electrospun PLLA films and solvent-cast glycine-polyvinyl alcohol (PVA) films. The results indicate that glycine-PCL nanofiber films exhibit superior ultrasound output efficiency, making them a preferred choice for implantable ultrasonic transducers and other biomedical actuation applications (Fig. 5e). Among these materials, PLLA exhibits the most balanced piezoelectric performance (~ 10–15 pC N^−1^) with moderate degradation rates (months to years), making it suitable for implantable devices. While PLA shares similar chemical structure with PLLA, its piezoelectric response is typically weaker due to lower crystallinity. In contrast, PCL offers excellent flexibility and slow degradation (years) but shows minimal piezoelectric activity (~ 1–5 pC N^−1^), limiting its use in energy harvesting applications. PVA, though highly biocompatible and tunable, requires physical/chemical modifications to achieve functional piezoelectricity and degrades rapidly in aqueous environments. In addition to biodegradable piezoelectric materials, conductive polymers play a crucial role in the development of stretchable and biocompatible electrodes for ultrasonic applications. Conventional metallic electrodes, though highly conductive, suffer from mechanical limitations, such as cracking and delamination under strain. Conductive polymers, including polythiophene (PTh), polyaniline (PANi), polypyrrole (PPy), and poly(3,4-ethylenedioxythiophene) polystyrene sulfonate (PEDOT:PSS), offer an alternative solution by combining high electrical conductivity with intrinsic mechanical flexibility [122]. Among these, PEDOT:PSS has attracted significant attention due to its high conductivity, biocompatibility, solution processability, and self-healing properties (Fig. 5f, g). While conductive polymers have been extensively employed in applications such as supercapacitors and sensors, their use in flexible medical imaging devices remains relatively underexplored. However, their stretchability and biocompatibility highlight their potential for integration into wearable and implantable ultrasonic transducers. Beyond conductive polymers, liquid metals have also been explored as highly conductive and stretchable alternatives. Due to their flowability, liquid metals can sustain extreme strains of up to 800% without mechanical failure [123]. These materials can be integrated into ultrasound devices by filling soft microchannels or micro-troughs to form conductive pathways. Additionally, liquid metals can be printed directly onto flexible substrates or mixed into elastomer matrices, such as PDMS, to create dynamically conductive and self-healing electrodes [124, 125]. However, the primary challenge associated with liquid metals in medical applications is their toxicity, as common liquid metal alloys, such as eutectic gallium-indium (EGaIn) and Galinstan, contain potentially harmful elements [126]. This limitation underscores the necessity of alternative stretchable conductive materials that are both biocompatible and mechanically robust.

### Energy Management

Efficient energy management is crucial for the successful application of flexible wearable ultrasound devices, which are typically intended for continuous physiological monitoring or long-term therapeutic interventions. Such applications impose significant challenges on energy supply, particularly given the devices’ requirements for lightweight and miniaturized designs. Traditional battery solutions often suffer from limitations in size, weight, and endurance, making efficient energy harvesting, transmission, and utilization pivotal to practical usability.

**Fig. 6:**
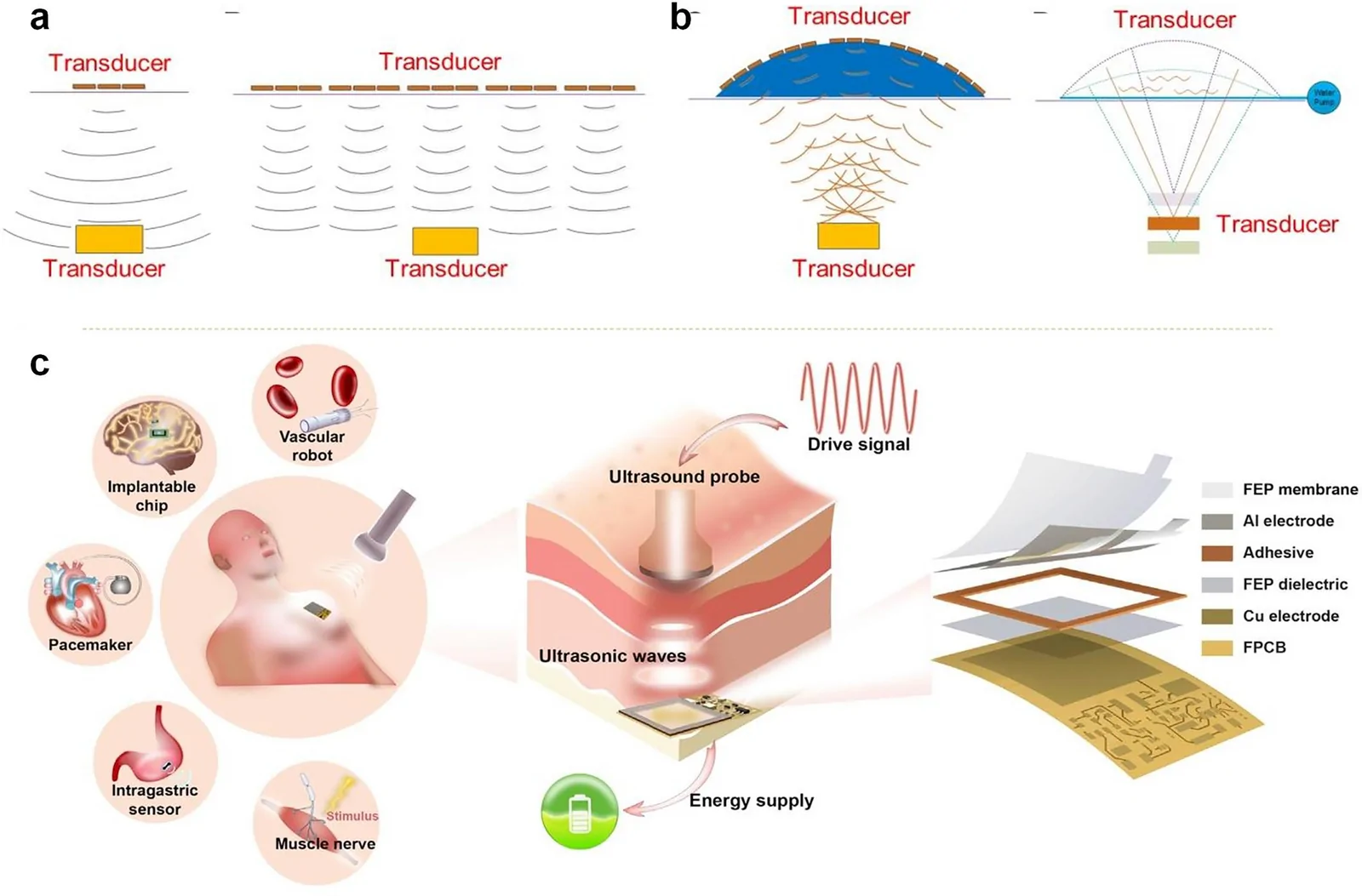
**a** Ultrasonic transmission using a few ultrasonic transducers.Ultrasonic transmission by a tiled array of ultrasonic transducers. Reproduced with permission [97]. Copyright 2021, AAAS. **b** Ultrasonic transmission by an array of ultrasonic transducers placed inside the top part of a flexible base. Reproduced with permission [97]. Copyright 2021, AAAS. **c** USD-TENG system is implanted under the skin and receives wireless energy from ultrasound to power the implanted IMDs. Reproduced with permission [131]. Copyright 2022, Elsevier Inc

Wireless power transfer, such as inductive power transfer, radiofrequency (RF) irradiation, acoustic power transfer (APT) and so on, presents a promising alternative power supply approach for flexible wearable biomedical sensors [127]. This technique enables device charging without physical connections, thereby enhancing convenience and durability. Inductive wireless power transfer is common in medical devices but struggles to balance small size with strong energy transfer, as it relies on tightly coupled coils [128]. RF methods can send power through tissue but suffer from absorption and require large receivers to capture enough energy [129, 130]. In contrast, ultrasound waves have lower tissue attenuation and shorter wavelengths, allowing for deeper operation with smaller devices. APT is a good choice for implantable devices, enables smaller, more efficient designs. The devices enable transfer via an acoustic method, converting mechanical energy into electrical energy (Fig. 6a, b) [97]. However, these charging exhibits constraints such as limited transmission distances and sampling frequencies, and existing rigid integrated circuit chips used for signal acquisition and wireless transmission further restrict overall system flexibility. Despite these limitations, wireless charging continues to hold significant potential for powering flexible wearable devices.

Energy harvesting technologies represent another critical strategy for sustainable energy supply in flexible wearable ultrasound devices, capturing energy from environmental sources or device-related mechanical movements to reduce or eliminate reliance on external power or frequent battery replacements. Acoustic energy harvesting, though relatively underexplored, holds promise by converting acoustic waves generated by the devices themselves or ambient sound sources into supplemental electrical energy. Moreover, triboelectric nanogenerators (TENGs) effectively convert mechanical energy derived from skin movements or pressure on transducers into electrical energy, enabling wireless, autonomous powering of wearable devices. Notably, research has demonstrated the viability of using external ultrasound waves to drive implantable triboelectric transducers, underscoring ultrasound as a viable direct energy source for implantable devices [131]. The capability of TENGs for self-powered operation has also been illustrated in wearable acoustic sensing systems, highlighting their significant potential in energy harvesting and autonomous operation (Fig. 6c).

Reducing power consumption is a crucial strategy for extending the operational duration of flexible wearable ultrasound devices. Low-power design approaches are particularly critical for ultrasound-on-chip platforms, which must achieve both substantial miniaturization and reduced energy consumption. Devices intended for continuous monitoring inherently require optimized energy management strategies that prioritize minimal power consumption while maximizing energy efficiency. Additionally, the energy conversion efficiency of ultrasound transducers plays a pivotal role in overall power consumption. Higher transducer efficiency reduces the electrical energy input required to achieve the same acoustic output, thereby improving overall device sustainability. As a result, developing high-efficiency ultrasound transducers remains a primary research focus for enhancing wearable device performance. Beyond transducer efficiency, material selection and sensing mechanisms significantly impact energy consumption in flexible wearable ultrasound systems. Comparative assessments of sensor sensitivity and power density further highlight the influence of material choices on energy management. Thus, carefully selecting appropriate materials-such as polymers or ceramic composites with superior piezoelectric properties-and designing efficient sensing mechanisms are critical to optimizing energy efficiency. These design choices collectively contribute to minimizing overall power consumption while maintaining reliable functionality in wearable ultrasound systems.

### Communication and Control Technology

Wearable ultrasound sensors are emerging as a key technology for continuous physiological monitoring, enabling real-time assessment of biomedical parameters. These flexible, skin-conformal devices noninvasively collect signals, which must be wirelessly transmitted to external devices for real-time data processing, medical analysis, and remote monitoring. Reliable wireless data transmission is essential for ensuring continuous health tracking, early disease detection, and integration with telemedicine platforms.

**Fig. 7:**
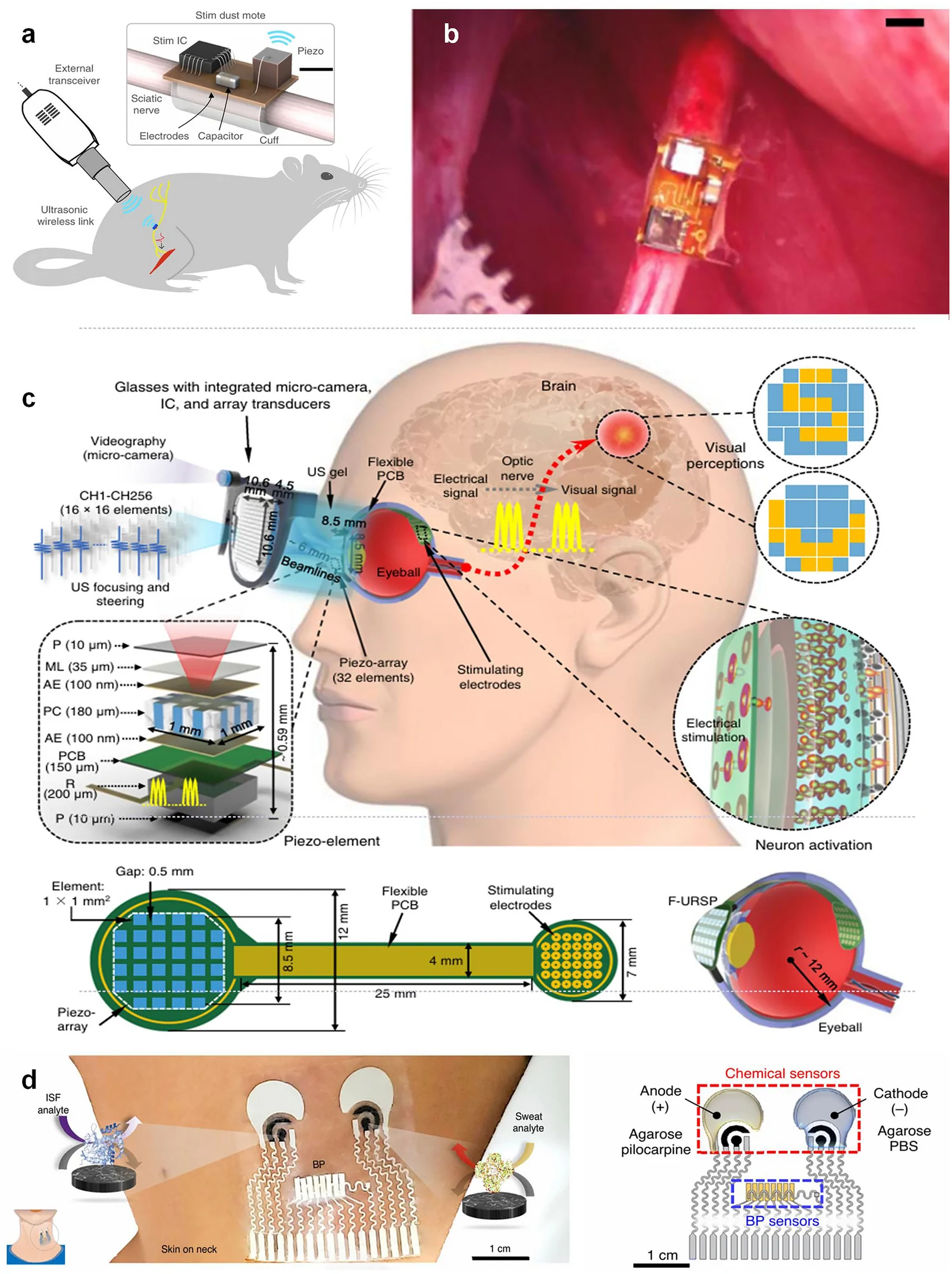
**a** Diagram of the StimDust system. Here it is used to stimulate the sciatic nerve of a rat. Scale bar. Reproduced with permission [133]. Copyright 2020, Springer Nature. **b** Stimulating mote fully implanted and affixed to a rat sciatic nerve. Reproduced with permission [133]. Copyright 2020, Springer Nature. **c** Schematic diagram showing the flexible ultrasonic device, with key components and aspect ratios labeled. Reproduced with permission [36]. Copyright 2022, Springer Nature. **d** Illustrations of the placement of the sensor and the enzymatic chemical sensors for ISF and sweat. Reproduced with permission [134]. Copyright 2021, Springer Nature

For extracorporeal physiological monitoring, such as ultrasound-based cardiovascular assessments, flexible wearable ultrasound patches have been developed to continuously collect vital signs [100]. A notable example is a soft ultrasound patch for continuous blood pressure monitoring, which provides a non-invasive, cuff-free alternative for evaluating arterial stiffness, central blood pressure, and cardiac output dynamics. The real-time data acquired by such sensors must be transmitted wirelessly to mobile health applications, cloud-based monitoring systems, or hospital networks, allowing healthcare professionals or AI-driven diagnostic algorithms to analyze physiological trends and detect anomalies [132]. In addition to traditional wireless methods, ultrasound communication has been explored as a means of data transmission and power delivery in biomedical applications, particularly for deeply implanted devices. David et al. introduced a novel wireless, wire-free, and miniaturized implantable neurostimulator called StimDust. Measuring only 1.7 cubic millimeters, this device utilizes ultrasound for wireless power transfer and bidirectional communication, overcoming the limitations of traditional implantable stimulators that require bulky batteries and wires (Fig. 7a, b) [133]. A new flexible ultrasound-powered retinal stimulation device has been developed as a wireless bionic visual prosthesis for the blind. It uses ultrasound to transmit energy and data, avoiding the risks and limitations of wired connections or electromagnetic transmission in traditional prostheses (Fig. 7c) [36]. Researchers demonstrated its ability to stimulate patterns in ex-vivo mouse retina and confirmed its good biocompatibility. This technology paves the way for safer and more efficient wireless artificial vision systems.

To support continuous, energy-efficient data transmission, wearable ultrasound sensors commonly utilize Bluetooth Low Energy (BLE). BLE is particularly advantageous for long-term ambulatory health monitoring because it provides low power consumption, moderate data transfer speeds, and reliable short-range communication (up to 10–30 m). Advanced multi-sensor wearable health platforms are now incorporating ultrasound blood pressure sensors alongside interstitial fluid (ISF) and sweat biosensors, allowing for a comprehensive, multimodal approach to health monitoring (Fig. 7d) [134]. In these integrated systems, all biomechanical, biochemical, and physiological data are wirelessly transmitted for centralized analysis, providing holistic insights into cardiovascular function, metabolic health, and hydration status. The ability to wirelessly synchronize multi-sensor data enhances personalized medicine, continuous disease monitoring, and AI-driven health analytics. 1By enabling continuous, real-time physiological monitoring, wireless ultrasound sensing technology reduces the burden of frequent hospital visits, supports early-stage disease intervention, and enhances longitudinal health tracking. Future innovations in ultra-low-power wireless chips, flexible antennas, and edge-computing health systems will further improve signal reliability, energy efficiency, and real-time data analytics, for next-generation bio-integrated wearable ultrasound devices.

## Applications of Wearable Ultrasound Devices in Therapy

One of the most promising applications of wearable ultrasound is drug delivery, where ultrasound waves can enhance the penetration of therapeutic agents, improving bioavailability and enabling targeted delivery. In tissue repair and regeneration, ultrasound has been shown to accelerate wound healing, promote bone growth, making wearable devices a compelling tool for noninvasive rehabilitation [135, 136]. The field of neuromodulation has also seen rapid advancements with ultrasound-based approaches for stimulating neural activity, offering potential treatments for neurological disorders such as chronic pain, depression, and movement disorders [137–139]. The following sections explore the key therapeutic areas where wearable ultrasound is making a profound impact, highlighting both current advancements.

### Drug Delivery

Ultrasound-mediated drug delivery enhances therapeutic efficacy by improving tissue penetration and overcoming biological barriers. Wearable ultrasound devices enable localized, repeatable delivery through mechanisms like cavitation and increased membrane permeability, allowing drugs to reach target tissues more effectively. Compared to systemic administration, this approach minimizes side effects and improves precision. Wearable systems are particularly suited for chronic conditions requiring frequent dosing, and are evolving into personalized, on-demand platforms that enhance both treatment outcomes.

**Fig. 8:**
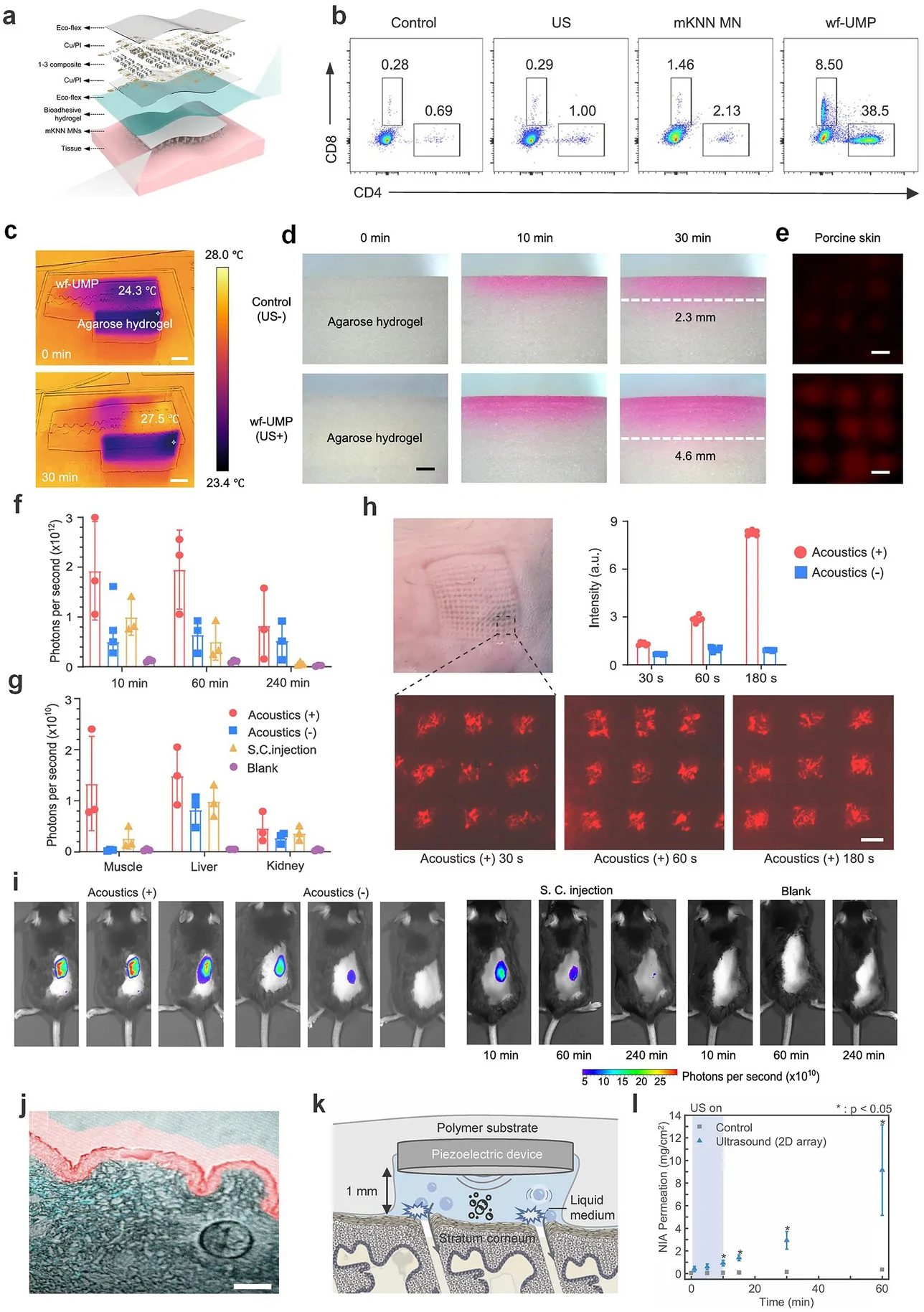
**a** Schematic of the integrated system level wf-UMP electronics, consisting of a flexible US transducer array for effective US emission, a bioadhesive hydrogel elastomer for robust adhesion and acoustic coupling layer, and a mKNN PNPs-loaded MN patch for drug delivery. Reproduced with permission [140]. Copyright 2025, Springer Nature. **b** FCM results of CD3 + CD8 + T cells and CD3 + CD4 + T cells infiltrated in tumors. Reproduced with permission [140]. Copyright 2025, Springer Nature. **c** Thermal images of wf-UMP on an agarose hydrogel operated continuously for 30 min. Top, 0 min. Bottom: 30 min. Scale bars, 1 cm. Reproduced with permission [141]. Copyright 2023, Springer Nature. **d** Comparison of drug delivery perfor mance of wf-UMP on agarose hydrogels with/without US stim ulation. Scale bars, 2 mm. Reproduced with permission [141]. Copyright 2023, Springer Nature. **e** Confocal microscopy images of porcine skin treated with Rho B labeled wf UMP with/without US stimulation at a depth of 328 µm. Scale bars, 250 µm. Reproduced with permission [141]. Copyright 2023, Springer Nature. **f** Quantifica tion of in vivo RhB dye release using the different methods. Reproduced with permission [141]. Copyright 2023, Springer Nature. **g** Presence of RhB dye in mouse muscle, liver, and kidney 24 h after the indicated treatments. Scale bar, 200 µm. Reproduced with permission [141]. Copyright 2023, Springer Nature. **h** After acoustic metamaterial treatments, representative images of mouse skin indi cated acoustic-mediated RhB dye delivery for 30, 60, or 180 s (fluorescent images). Reproduced with permission [141]. Copyright 2023, Springer Nature. **i** IVISR images of mice post dye delivery (10, 60, or 240 mvia acoustic metamaterials methods. Reproduced with permission [141]. Copyright 2023, Springer Nature. **j** Multiphoton confocal microscopy image showing RhB (rhodamin penetration into a vertical Sect. (7 μm thi of a porcine skin sample following passive diffusion; scale bar: 200 μm. Reproduced with permission [88]. Copyright 2023, Wiley–VCH GmbH. **k** Schematic illustration of the cUSP on skin, showing the cavitation mechanism within the cavity between the device and the skin, and resulting drug penetration through stratum corneum. Reproduced with permission [88]. Copyright 2023, Wiley–VCH GmbH. **l** Amount of NIA permeated with application of ultrasound versus control (passive permeafor the cUSP array. Reproduced with permission [88]. Copyright 2023, Wiley–VCH GmbH

Xue et al. evaluated the transdermal drug delivery performance of a wearable flexible ultrasound microneedle patch (wf-UMP) that integrates lead-free piezoelectric ultrasound transducers and dissolvable microneedles (MNs) loaded with piezocatalytic nanoparticles (Fig. 8a) [140]. Figure 8b, c illustrates the time-dependent diffusion of Rhodamine B (RhB), a model drug, from the dissolvable MNs into the hydrogel. The diffusion region expanded significantly over time, and was markedly enhanced under ultrasound stimulation (US +), compared to the non-ultrasound condition (US −), indicating that ultrasound facilitates both drug release and penetration into the surrounding tissue-mimicking matrix. Upon application to agarose hydrogels, the dissolvable MNs exhibited excellent solubility and rapid drug release. Comparative studies showed that ultrasound (US) stimulation significantly enhanced drug diffusion, doubling the penetration depth from ~ 2.3 mm (US −) to ~ 4.6 mm (US +) at 30 min (Fig. 8d). Further validation on porcine skin revealed an increased fluorescence intensity and a ~ 500 μm diffusion depth in US + samples compared to < 400 μm in US– controls. This enhancement was attributed to US-facilitated diffusion of Rhodamine B dye and activation of mKNN PNPs released from the MN tips (Fig. 8e), confirming the potential of wf-UMP as an effective transdermal drug delivery platform for cancer therapy.

In their evaluation, Xu et al. developed an innovative acoustic metamaterial-mediated patch system for transdermal drug delivery, aimed at overcoming the limitations of conventional needle-based injections and passive delivery (Fig. 8f) [141]. The authors integrated sharp pyramid-shaped acoustic metamaterial structures into a therapeutic patch that could both mechanically penetrate the skin and actively transport therapeutics through localized acoustic streaming. In vitro and ex vivo studies using Rhodamine B (RhB) as a model compound demonstrated significantly enhanced dye release with acoustic stimulation compared to passive diffusion, with a 9.3-fold increase in transdermal delivery within the same treatment period (Fig. 8g, h). In vivo tests further revealed superior dye retention and biodistribution when compared to subcutaneous injections (Fig. 8i). As a proof-of-concept, this programmable patch enabled dynamic, multi-burst delivery of epinephrine to reverse anaphylaxis in mice, outperforming the traditional fixed-dose epinephrine injection strategy. The platform’s programmable dose release and improved pharmacokinetic control highlights its promising utility in personalized, on-demand management of acute diseases. Moreover, Meng et al. used MR-guided focused ultrasound (MRgFUS) to deliver the monoclonal antibody trastuzumab in four patients with Her2-positive brain metastasis [142]. The treatment was safe and increased drug delivery into MRgFUS targeted compared to nontargeted lesions. This first-in-human trial suggests that MRgFUS is a safe and effective method to deliver treatments across the blood–brain barrier (BBB) and paves the way for the use of this method for other neurological conditions. Yu et al. explored a conformable ultrasound (US) patch based on a 2D array of piezoelectric transducers embedded in a soft polymer substrate, aiming to enhance transdermal delivery through cavitation [88]. As illustrated in Fig. 8j and k, the patch conforms closely to the skin surface, enabling effective acoustic coupling and inducing localized cavitation at the stratum corneum to transiently disrupt the barrier function. To quantify drug permeation, a Franz diffusion cell system was used, with porcine skin mounted between donor and receptor chambers, temperature maintained at 32 °C, and permeated niacinamide (NIA) detected via HPLC analysis. Permeation results (Fig. 8l) demonstrated that the US-treated group achieved a dramatic increase in NIA transdermal delivery—reaching 12.3 mg cm^−2^ at 60 min—compared to the control group, which remained below 1.0 mg cm^−2^ throughout. Statistically significant enhancement (*p* < 0.05) was observed as early as 10 min into US application, emphasizing the rapid onset and strong efficacy of acoustic stimulation. The enhanced transport was attributed to stable cavitation and acoustic streaming, which facilitated drug diffusion without notable thermal effects or skin damage. Li et al. developed a stretchable electronic facial mask (SEFM) to enhance transdermal drug delivery via ultrasound-induced sonophoresis [143]. The device integrated a piezoelectric ultrasonic array operating at 1 MHz within a conformable, facially fitted silicone encapsulation, aiming to facilitate drug penetration across the skin barrier. To assess its efficacy, rhodamine-labeled hyaluronic acid was applied onto the skin of Sprague–Dawley rats, followed by ultrasound treatment using the SEFM. Confocal microscopy revealed significantly increased fluorescence intensity at depths of 0–15 μm in the ultrasonic group versus controls (*p* < 0.05), indicating deeper and more effective drug permeation. Further, in human trials, SEFM application resulted in an average 20% increase in facial skin moisture compared to the control group, supporting enhanced delivery of HA. The experiment was extended to other facial drugs—2% beta-glucan and 2% D-panthenol—and results consistently showed deeper skin deposition in the ultrasound-treated group, verified via fluorescence imaging of skin slices. These findings underscore the potential of ultrasound-assisted stretchable electronics in non-invasive, efficient transdermal drug delivery platforms, particularly for macromolecular therapeutics in cosmetic and dermatologic applications. Collectively, these findings validate the utility of soft ultrasound electronics in enabling non-invasive, efficient, and spatially controllable delivery of both cosmetic and therapeutic agents across the skin barrier.

### Tissue Repair and Regeneration

Wearable ultrasound systems offer a unique approach to promoting tissue repair and regeneration by enabling continuous, localized, and non-invasive mechanical stimulation. Through soft, skin-conformal designs, these devices can deliver low-intensity ultrasound to enhance cell proliferation, angiogenesis, and matrix remodeling-key processes in healing injured tissuesPowles. Their portability and compatibility with daily activities make them especially valuable for long-term, personalized regenerative therapies.

**Fig. 9:**
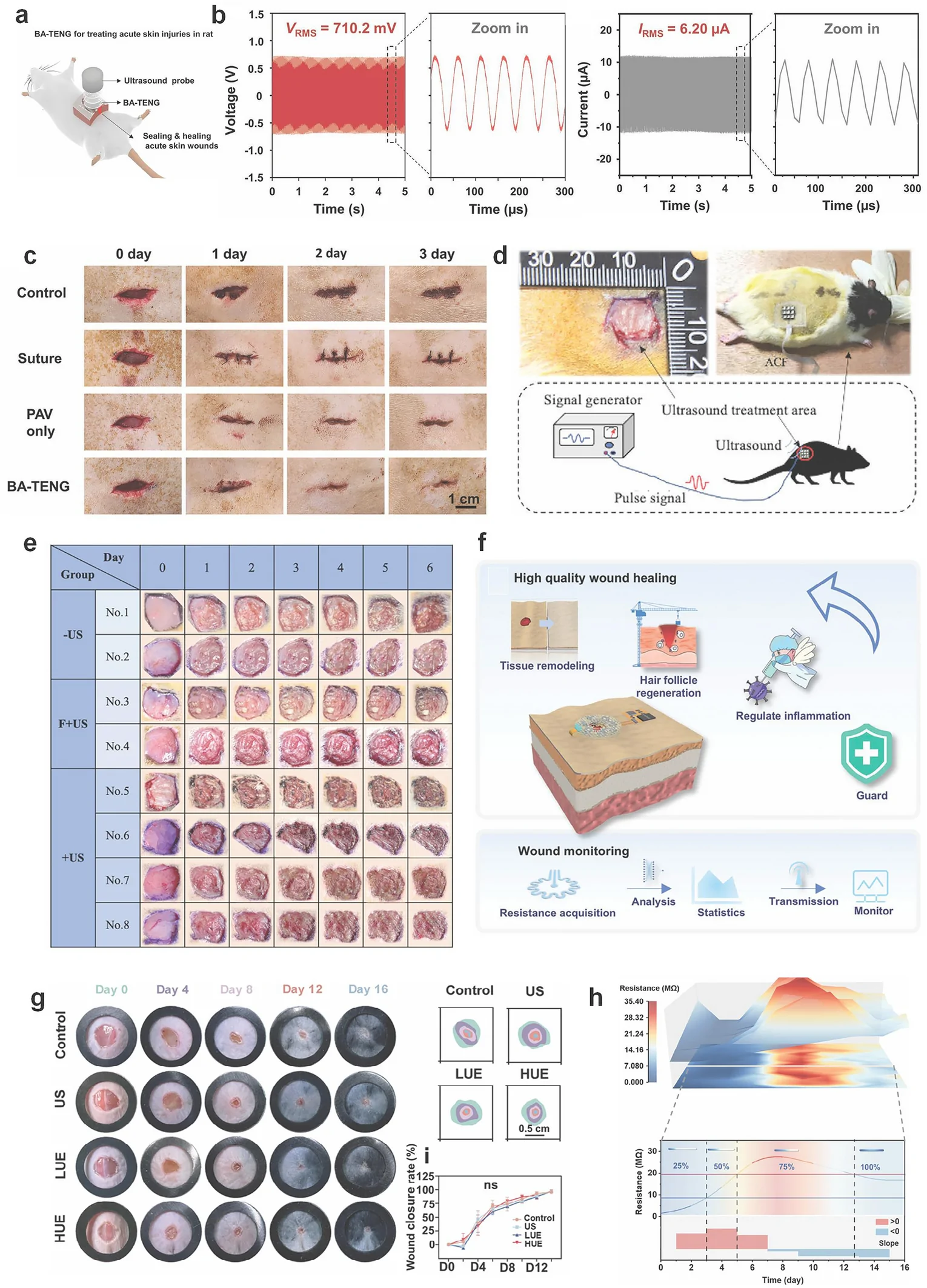
**a** Schematic of the BA-TENG in sealing and ultrasound driven electrically healing rat skin injuries. Reproduced with permission [144]. Copyright 2023, Wiley–VCH GmbH. **b** Voltage and current output of the BA-TENG measured in water at 5∼mm from the ultrasound probe under 1∼W cm^−2^. Reproduced with permission [144]. Copyright 2023, Wiley–VCH GmbH. **c** Comparison of 3-day wound healing images in the untreated (contsuture, bioadhesive PAV only, and BA-TENG treated groups. Scalebar:1∼cm. Reproduced with permission [144]. Copyright 2023, Wiley–VCH GmbH. **d**Schematic diagram of wound treatment with the flexible ultrasonic patch. Reproduced with permission [90]. Copyright 2021, Wiley–VCH GmbH. **e** Healing states of the wound surface during treatment for the eight groups. Reproduced with permission [90]. Copyright 2021, Wiley–VCH GmbH. **f** Conceptual illustration of multifunctional UEP in high-quality wound healing and monitoring. Reproduced with permission [145]. Copyright 2025, Wiley–VCH GmbH. **g** Optical images of wound closure collected on days 0, 4, 8, 12, and 16 for the control, US, LUE, and HUE groups. Reproduced with permission [145]. Copyright 2025, Wiley–VCH GmbH. **h** 3D color mapping of skin–wound resistance trends (named: recovery cur. The prediction of wound monitoring and healing time under the recovery curve. Reproduced with permission [145]. Copyright 2025, Wiley–VCH GmbH

In their study, Meng et al. developed a bioadhesive triboelectric nanogenerator (BA-TENG) for rapid wound sealing and ultrasound-driven electrical stimulation to accelerate healing [144]. The BA-TENG, composed of biocompatible materials with a flexible TENG layer atop a bioadhesive interface, demonstrated high interfacial toughness (~ 150 J m⁻^2^) and rapid adhesion (~ 5 s) even on wet tissue. In a rat bleeding liver incision model, the BA-TENG achieved instant hemostasis and reduced blood loss by approximately 82%. The therapeutic protocol involved applying the BA-TENG onto the skin wound followed by activation with an ultrasound probe (20 kHz, 1 W cm⁻^2^), as illustrated in a rat model of acute injury (Fig. 9a). Upon ultrasound exposure, the device generated a stable voltage of 1.42 V and a current of 22.0 µA, producing a serrated electric field of ~ 0.86 kV m⁻^1^ across the wound interface (Fig. 9b). This field facilitated enhanced fibroblast migration and proliferation, leading to significantly accelerated wound healing. Compared to control, the BA-TENG group showed superior wound closure kinetics, with near-complete re-epithelialization observed within 3 days (Fig. 9c), thereby demonstrating the synergistic benefits of ultrasound-triggered electrical stimulation in promoting tissue regeneration.

Similarly, Lyu et al. proposed a flexible ultrasonic patch engineered for chronic wound therapy, composed of PZT-4 piezoelectric ceramic units arranged on an “island-bridge” flexible substrate, encapsulated with a hydrogel layer to enable conformal contact and prevent infection[90]. In the treatment protocol, the patch was affixed over full-thickness wounds in diabetic rats and subjected to daily 20-min ultrasound stimulations (6 V input, ~ 20 mW cm⁻^2^ acoustic intensity). Figure 9d schematically illustrates the application of the patch onto the wound site with embedded ultrasound units enabling targeted mechanical stimulation. Over the course of one week, rats receiving ultrasound treatment exhibited significantly faster wound healing. The progressive wound closure and superior healing state in the + US group are clearly demonstrated in Fig. 9e, affirming the patch’s therapeutic efficacy in reversing fibroblast senescence and accelerating tissue repair.

More recently, Chen et al. introduced a fully integrated ultrasonic–electric-coupled patch (UEP), designed to provide full-space, multimodal stimulation and real-time monitoring for wound regeneration [145]. The patch synergistically combined an ultrasonic array and an electric stimulation coil, enabling simultaneous delivery of deep-penetrating acoustic waves and surface-directed electric fields. As illustrated in the right portion of Fig. 9f, this configuration allowed the ultrasound to target deeper wound layers, while the electric field remained concentrated at the surface, promoting fibroblast activity and inflammation modulation across tissue depth. In vivo wound healing studies on full-thickness murine dorsal skin demonstrated the efficacy of the UEP, where animals treated with high-intensity ultrasonic–electric stimulation (HUE) exhibited visibly smaller wound areas over time compared to control, ultrasound-only (US), or low-intensity (LUE) groups (Fig. 9g). Notably, the authors introduced a novel resistance-based wound monitoring strategy, whereby changes in skin–wound impedance were mapped into a 3D “recovery curve.” As shown in Fig. 9h, different resistance ranges corresponded to distinct healing phases (inflammatory, proliferative, remodeling), enabling predictive assessment of wound healing trajectory and stage classification with machine learning assistance. Liu et al. demonstrated a stretchable ultrasound array specifically tailored for bone fracture healing [146]. The system employed high-performance 1–3 piezoelectric composites as transducers, stretchable serpentine metal interconnects, and PDMS-based adhesive layers, integrated onto soft elastomeric membranes to ensure conformal attachment to skin. Overall, ultrasound-enabled wearable devices have demonstrated strong potential in promoting tissue regeneration by modulating cellular behaviors such as proliferation, migration, and differentiation. Whether through mechanical stimulation, electric coupling, or multimodal integration, these systems effectively enhance wound healing and tissue remodeling across both soft and hard tissues.

### Neuromodulation

Ultrasound can penetrate soft tissues without direct contact with nerves, allowing modulation of neural activity through mechanical effects such as acoustic radiation force or cavitation. Offering a promising strategy for treating neurological disorders, enhancing neuroplasticity, and supporting functional recovery after injury.Ultrasound can noninvasively stimulate neural tissue by inducing mechanical pressure waves that activate mechanosensitive ion channels in neurons [147, 148]. This technique, known as ultrasonic neuromodulation, can reach deep neural targets without surgery or implants. An example is a wearable auricular vagus nerve stimulator in the form of a headset, which presses an ultrasound transducer against the ear to stimulate the vagal nerve branch [149]. This ultrasonic headset is compact and shown to effectively induce calm by activating vagal pathways. Compared to implanted electrical vagus nerve stimulators, a wearable ultrasound device offers a safer, non-invasive alternative with easy application and removal.

**Fig. 10:**
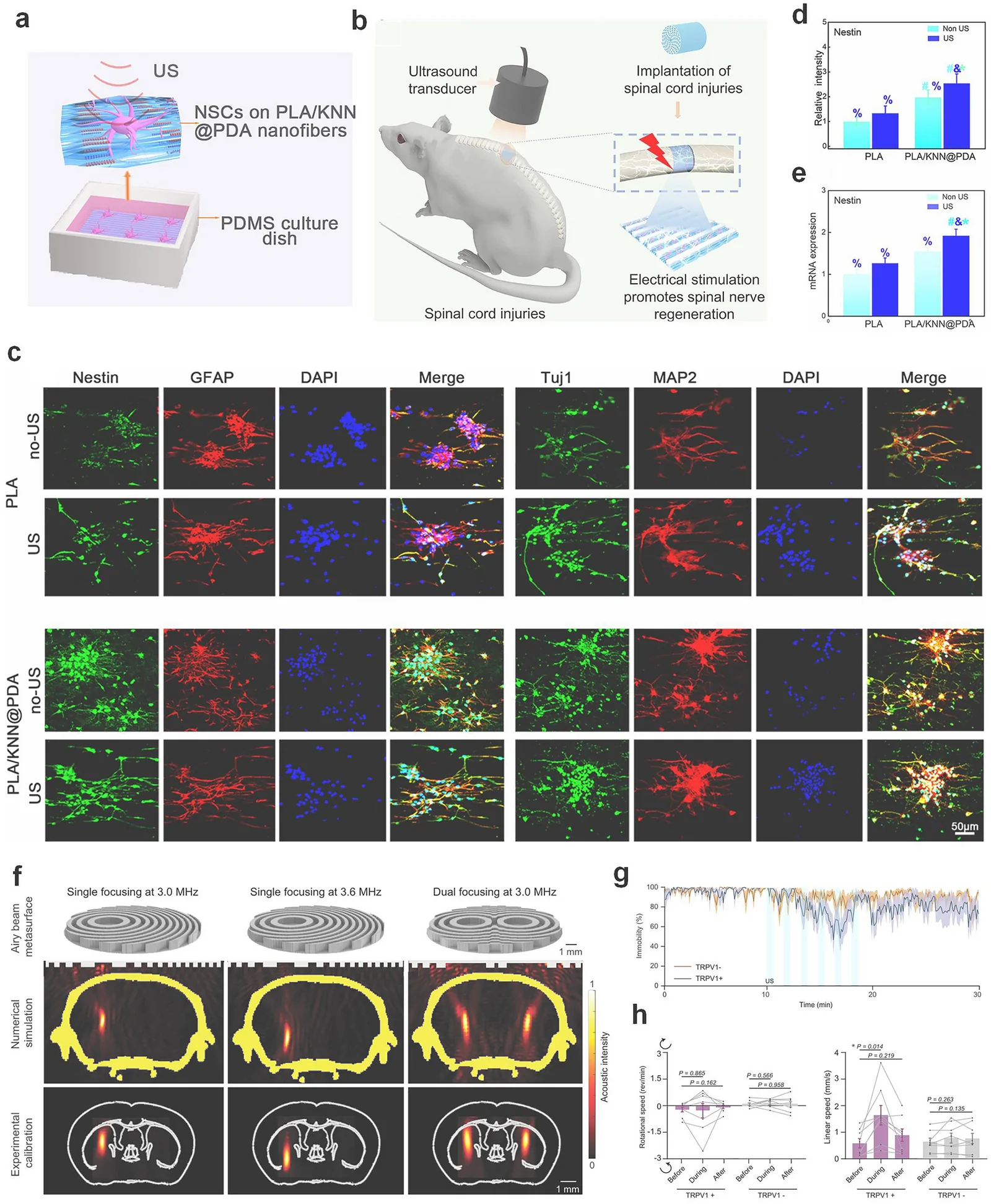
**a** Schematic diagram of the piezoelectric nanofiber scaffolds combining US promoted NSCs differentiation. Reproduced with permission [150]. Copyright 2022, ACS Nano. **b** Ultrasound powered wireless ES based on a biodegradable high-performance 3D piezoelectric scaffold promotes neural regeneration in spinal cord injuries. Reproduced with permission [150]. Copyright 2022, ACS Nano. **c** Immunofluorescent stain ing of the early neural markers Nestin and β-Tubulin III (Tuj1), the later neural marker MAP2, and neurogliocyte specific maker glial fibrillary acidic protein (GFAP) after 7 days culture. Reproduced with permission [150]. Copyright 2022, ACS Nano. **d** Statistical analysis of the f luorescence intensity of Nestin. Reproduced with permission [150]. Copyright 2022, ACS Nano. **e** Relative mRNA expression of four proteins. Reproduced with permission [150]. Copyright 2022, ACS Nano. **f** Off-center beam steering, dynamic beam steering along the wave- propagation direction by adjusting the ultrasound frequency without altering the metasurface, and dual focusing. Reproduced with permission [151]. Copyright 2024, PNAS. **g** Assessment of mouse immobility, quantified as the percentage of time spent immobile before, during, and after ultrasound stimulation. Reproduced with permission [151]. Copyright 2024, PNAS. **h** Observed enhancement in motor activity demonstrates the therapeutic potential of bilateral AhSonogenetic stimulation in restoring function in neurodegenerative conditions. Reproduced with permission [151]. Copyright 2024, PNAS

In their evaluation of ultrasound neuromodulation, Wang et al. systematically demonstrated how sonopiezoelectric biomaterials-particularly potassium sodium niobate (KNN)-based nanofibers, which can enable noninvasive, ultrasound-triggered neural modulation through localized electrical stimulation [150]. As Fig. 10a illustrates the conceptual framework of this approach, where a biodegradable piezoelectric scaffold is implanted at the site of spinal cord injury. Upon transcutaneous ultrasound stimulation, the scaffold generates localized electric fields that promote spinal nerve regeneration, offering a noninvasive strategy for neural repair. By depicting a setup in which neural stem cells (NSCs) are cultured on PLA/KNN@PDA (polylactic acid/polydopamine-coated KNN) nanofibers within a PDMS culture dish. When ultrasound is applied, the scaffold provides electrical stimulation to the NSCs, highlighting a controllable in vitro model of neuromodulation (Fig. 10b). This stimulation paradigm is further validated in Fig. 10c–e, which demonstrates both morphological and molecular changes in NSCs in response to ultrasound. Immunofluorescence images (Fig. 10c) show increased expression of Nestin (a marker for neural progenitor cells) and GFAP under ultrasound stimulation, particularly in the PLA/KNN@PDA condition. Quantitative analysis (Fig. 10d, e) confirms that ultrasound significantly upregulates Nestin expression at both the protein and mRNA levels, indicating enhanced neural differentiation and activation. Together, these findings provide compelling evidence for the application of ultrasound-activated piezoelectric scaffolds in neuromodulation and regenerative therapy.

Hu et al. introduced Airy-beam holographic sonogenetics (AhSonogenetics) as a transformative approach for achieving highly precise, flexible, and noninvasive neuromodulation in freely moving animals [151]. Central to this innovation is the use of 3D-printed metasurfaces that generate sharply focused Airy beams capable of targeting genetically sensitized neurons expressing ultrasound-responsive ion channels, such as TRPV1. Figure 10f depicts the finalized wearable ultrasound device developed for this purpose. The device integrates a planar PZT ultrasound element with a custom-designed metasurface enclosed in a miniaturized 3D-printed housing that attaches to a mouse’s skull. This compact, lightweight design enables stable and repeatable targeting of deep brain regions with submillimeter focal precision, a notable advancement over previous wearable ultrasound systems with large and imprecise focal zones. This configuration allows for dynamic beam steering by modulating the driving frequency, enabling depth-specific stimulation within a single brain hemisphere without replacing hardware components. Functionally, this capability is validated behavioral from a Parkinson’s disease mouse model receiving bilateral AhSonogenetic stimulation of the dorsal striatum (Fig. 10g). During ultrasound application, TRPV1-overexpressing (TRPV1⁺) mice showed a significant increase in linear locomotor speed-rising from 0.61 to 1.70 mm s^−1^, whereas control (TRPV1⁻) mice exhibited no significant behavioral change. The observed enhancement in motor activity demonstrates the therapeutic potential of bilateral AhSonogenetic stimulation in restoring function in neurodegenerative conditions (Fig. 10h).

Taken together, ultrasound neuromodulation represents a rapidly evolving frontier in neural engineering, offering a compelling combination of noninvasiveness, deep tissue penetration, and high spatial–temporal precision. By leveraging the mechanical energy of focused ultrasound to modulate neural excitability, this modality enables targeted stimulation of both superficial and deep brain structures without the need for surgical implants. Its compatibility with genetic, optical, and electrophysiological tools further broadens its utility across basic neuroscience research and translational medicine. As device engineering, sonogenetic actuators, and waveform strategies continue to advance, ultrasound is poised to become a core technology for precise, adaptive, and minimally invasive neuromodulatory therapies.

## Challenges and Limitations

Wearable ultrasound technology faces several critical challenges that must be overcome to realize its full clinical potential. Material durability remains a fundamental limitation, as traditional PZT piezoelectric materials provide high performance but are rigid and brittle, making them unsuitable for flexible applications [105, 152]. Piezoelectric polymers like polyvinylidene fluoride (PVDF) offer mechanical adaptability but suffer from lower electromechanical coupling efficiency, limiting their sensitivity and bandwidth [153]. Recent advances in 1–3 piezoelectric composites and nanomaterials have shown promise in bridging this performance gap, but challenges persist in maintaining polarization stability during repeated mechanical deformation and developing cost-effective, scalable fabrication methods. The mechanical interface with biological tissues presents another major hurdle. While stretchable phased-array designs allow electronic beam focusing and steering, they often increase the spacing between transducer elements, limiting spatial resolution at higher frequencies [34]. From a biological standpoint, prolonged skin contact raises concerns about dermatitis (reported in 15%–20% of users in extended wear studies), and implantable components must address potential inflammatory responses to degradation byproducts. Clinical translation faces unique validation challenges, including motion artifacts during continuous monitoring (reducing signal-to-noise ratio by up to 50% during normal activity) and the absence of regulatory frameworks for long-term use—current FDA guidelines for diagnostic ultrasound don’t adequately address the safety considerations of wearable applications involving 24/7 exposure. Potential solutions are emerging across these fronts: novel manufacturing techniques like aerosol jet printing enable high-resolution patterning of flexible transducer arrays, while machine learning algorithms have demonstrated 40%-60% improvement in motion artifact correction. Bioadhesive hydrogels with controlled hydration properties can maintain coupling efficiency for over 48 h, and hybrid energy harvesting systems may solve power limitations by combining piezoelectric and triboelectric effects. For biological compatibility, researchers are developing ultrasonic metagels that degrade predictably without provoking immune responses [154]. These interdisciplinary advances, coupled with ongoing efforts to establish wearable-specific regulatory standards, are paving the way for clinically viable solutions that maintain diagnostic/therapeutic efficacy while addressing the unique demands of continuous, patient-administered ultrasound applications.

## Future Directions

The future of wearable ultrasound hinges on advances in materials, device design, and smart integration. Novel piezoelectric nanocomposites and 2D materials like MXenes are enabling flexible “piezo skins” to replace rigid ceramics, while 3D printing allows patient-specific patches for better acoustic coupling. Device architectures are evolving toward miniaturized, multifunctional systems with embedded beamforming chips and adaptive feedback (e.g., motion-compensated arrays). AI and IoT are critical for real-time analysis, enabling personalized therapy adjustments and remote monitoring through cloud-connected platforms. For commercialization, standardization and scalable manufacturing are key challenges. Roll-to-roll printing and injection molding could lower costs, while clinical trials must validate efficacy for regulatory approval. Cross-disciplinary collaboration will ensure seamless integration into healthcare systems, bridging the gap from lab to clinic.

While wearable ultrasound has demonstrated significant potential in drug delivery, tissue regeneration, and neuromodulation, its applications are rapidly expanding into other critical medical domains through technological advancements. These devices now enable continuous, non-invasive sensing technology of physiological vibrations with sub-micron resolution, such as using deep learning algorithms to correlate the single-transducer radio-frequency data from forearm muscles with hand gestures to accurately and continuously track 13 hand joints with a mean error of only 7.9° [155]. Furthermore, the development and testing of a prototype skin-conformal ultrasonic phased array can realize the monitoring of hemodynamic signals from tissues up to 14 cm beneath the skin. The device allows for active focusing and steering of ultrasound beams over a range of incident angles so as to target regions of interest [156]. Beyond monitoring, the bioadhesive ultrasound device that consists of a thin ultrasound probe robustly adhered to the skin via a couplant made of a soft, tough, antidehydrating, and bioadhesive hydrogel-elastomer hybrid can provides 48 h of continuous imaging of diverse internal organs, including blood vessels, muscle, heart, gastrointestinal tract, diaphragm, and lung, which could enable diagnostic and monitoring tools for various diseases[154]. These developments underscore wearable ultrasound’s dual diagnostic-therapeutic capabilities, though challenges remain in establishing signal standardization protocols and adapting regulatory frameworks for continuous-use applications.

## Summary

Wearable ultrasound devices have emerged as a versatile and promising technology with the potential to transform therapeutic applications. Recent advancements have enabled their use in noninvasive drug delivery, accelerated tissue repair, targeted neuromodulation, and cancer therapy. These devices harness the well-established bioeffects of ultrasound, such as enhancing permeability, stimulating regeneration, and modulating neural activity, while integrating modern materials and electronics to create portable, patient-friendly solutions. Though still in early clinical trials, studies have demonstrated their ability to accelerate wound healing, improve bone fracture repair, enable noninvasive nerve stimulation, and suppress tumor growth, underscoring their therapeutic potential. The key advantage of wearable ultrasound lies in its ability to provide continuous or on-demand therapy noninvasively, improving treatment efficacy while enhancing patient comfort and compliance. However, significant challenges remain in material durability, device performance, clinical translation, and commercial scalability. Active research is addressing these issues, with interdisciplinary collaborations playing a crucial role in refining these technologies and ensuring they meet real-world clinical needs. Overcoming these barriers could lead to a paradigm shift in treatment strategies, such as managing chronic wounds with a smart patch at home or treating neurological disorders with a wearable neuromodulation cap as an alternative to medication. Addressing these barriers could pave the way for widespread adoption, particularly as wearable ultrasound aligns with broader trends in personalized medicine, remote healthcare, and AI-driven diagnostics, positioning it as a key player in future digital health ecosystems. Ultimately, wearable ultrasound is poised to revolutionize healthcare by enabling noninvasive, real-time, and personalized monitoring and therapy. Advancements in materials, device architecture, AI-driven optimization, and commercial scalability will be key to overcoming current limitations. As these technologies mature, continuous, wireless, and precise deep-tissue monitoring and treatment will transition from concept to widespread clinical reality, ushering in a new era of intelligent, patient-centric healthcare.
